# The Model of the Conserved Epigenetic Regulation of Sex

**DOI:** 10.3389/fgene.2019.00857

**Published:** 2019-09-26

**Authors:** Francesc Piferrer, Dafni Anastasiadi, Alejandro Valdivieso, Núria Sánchez-Baizán, Javier Moraleda-Prados, Laia Ribas

**Affiliations:** Institut de Ciències del Mar (ICM), Spanish National Research Council (CSIC), Barcelona, Spain

**Keywords:** conserved epigenetic regulation of sex, essential epigenetic marks, DNA methylation, sex determination, sex differentiation, sex control, environmental sex determination

## Abstract

Epigenetics integrates genomic and environmental information to produce a given phenotype. Here, the model of Conserved Epigenetic Regulation of Sex (CERS) is discussed. This model is based on our knowledge on genes involved in sexual development and on epigenetic regulation of gene expression activation and silencing. This model was recently postulated to be applied to the sexual development of fish, and it states that epigenetic and gene expression patterns are more associated with the development of a particular gonadal phenotype, e.g., testis differentiation, rather than with the intrinsic or extrinsic causes that lead to the development of this phenotype. This requires the existence of genes with different epigenetic modifications, for example, changes in DNA methylation levels associated with the development of a particular sex. Focusing on DNA methylation, the identification of CpGs, the methylation of which is linked to sex, constitutes the basis for the identification of Essential Epigenetic Marks (EEM). EEMs are defined as the number and identity of informative epigenetic marks that are strictly necessary, albeit perhaps not sufficient, to bring about a specific, measurable, phenotype of interest. Here, we provide a summary of the genes where DNA methylation has been investigated so far, focusing on fish. We found that *cyp19a1a* and *dmrt1*, two key genes for ovary and testis development, respectively, consistently show an inverse relationship between their DNA methylation and expression levels, thus following CERS predictions. However, in *foxl2a*, a pro-female gene, and *amh*, a pro-male gene, such relationship is not clear. The available data of other genes related to sexual development such as *sox9*, *gsdf*, and *amhr2* are also discussed. Next, we discuss the use of CERS to make testable predictions of how sex is epigenetically regulated and to better understand sexual development, as well as the use of EEMs as tools for the diagnosis and prognosis of sex. We argue that CERS can aid in focusing research on the epigenetic regulation of sexual development not only in fish but also in vertebrates in general, particularly in reptiles with temperature sex-determination, and can be the basis for possible practical applications including sex control in aquaculture and also in conservation biology.

## Introduction

### Background on Epigenetics

The origin of the term “epigenetics” and its implications are continuously subjected to revision. Here, we will use the definition proposed by Deans and Maggert (2015): “the study of phenomena and mechanisms that cause chromosome-bound, heritable changes to gene expression that are not dependent on changes to DNA sequence.” These epigenetic changes or epimutations can be inherited not only during mitosis from mother to daughter cells but also through meiosis from parents to offspring (Dupont et al., 2009). Epigenetics has emerged as a powerful discipline in the study of the integration of genomic and environmental information to bring about a specific phenotype (Turner, 2009; Vogt, 2017).

Fish sex is remarkably plastic when compared with the situation in other vertebrates since it can be determined genetically, environmentally, or by a combination of both types of influences (see Wang et al., 2019 and articles therein). Fish present three major sexual patterns: gonochorism, hermaphroditism, and unisexuality. Thus, the phenotypic sex is, in many fish, a clear example of phenotypic plasticity not only because, in hermaphrodites, the same genotype is capable of producing two different phenotypes but also because, under certain environmental conditions, e.g., unusually warm temperatures, some gonochoristic species may develop a phenotypic sex different from its genotypic sex (Ospina-Alvarez and Piferrer, 2008; Baroiller and D’Cotta, 2016; Ribas et al., 2017).

During sexual differentiation, cells of the germ and somatic lines acquire identity and, in this process, changes in gene expression patterns play a central role. Thus, sexual differentiation involves a certain antagonism between male and female pathways as well as multiple feedback loops that reinforce the effects of the primary effector, be genetic or environmental (Munger and Capel, 2012). Gene networks, involved in testis or ovarian differentiation, consist of genes the expression of which is activated or suppressed in a tight spatial and temporal fashion (Capel, 2017). We now know that in this type of regulation, epigenetic mechanisms such as DNA methylation, histone modification, and noncoding RNAs (Berger et al., 2009) play a role, and hence, in the last years, the contribution of epigenetics to sex determination and differentiation across taxa has emerged (reviewed in Piferrer, 2013). In the rest of this paper, we will use the term “sexual development” when collectively referring to sex determination and sex differentiation.

### The Model of Conserved Epigenetic Regulation of Sex

Recently, the concept of Essential Epigenetic Marks (EEM), defined as “the number and identity of informative epigenetic marks that are strictly necessary, albeit perhaps not sufficient, to bring about a specific, measurable, phenotype of interest,” was proposed (Piferrer, 2019). The model of Conserved Epigenetic Regulation of Sex (CERS) was also proposed (Piferrer, 2019) in regards to the regulation of gene expression during the emergence of the sexual phenotype. This model is based on the assumptions that there are “pro-male” and “pro-female” genes and that there is an inverse relationship between epigenetic silencing and expression of the genes. The terms “pro-male” and “pro-female” genes refer to the exclusive or preferential expression of these genes in one sex rather than in the other. Specifically, the model applies to sex differentiation in gonochoristic species and sex change in hermaphroditic species regardless of the underlying sex-determining mechanism. The CERS model postulates that, for a given sex-related gene, the association between DNA methylation and expression levels with a particular gonadal phenotype is stronger than the means by which this phenotype is obtained (Piferrer, 2019). This implies that, in females, DNA methylation of pro-female genes will be low while expression of these genes will be high and that, in contrast, DNA methylation of pro-male genes will be high while their expression will be low. Conversely, in males, DNA methylation of pro-male genes will be low, while expression of these genes will be high and, in contrast, DNA methylation of pro-female genes will be high, while their expression will be low. Notice that “low” and “high” rather than absolute values indicate values of one sex relative to the other sex. The regulation of gene expression levels by changes in DNA methylation constitute one of the main molecular mechanisms of CERS (the other two would be regulation of gene expression by histone modifications or variants and abundance and activity of miRNAs).

Regarding the causation of differentially methylation levels of “pro-male” and “pro-female” genes, currently, there is debate on whether epigenetic changes are a cause or a consequence of changes in gene expression (probably both things are correct). Allele-specific effects have been found in the half-smooth tongue sole, *Cynoglossus semilaevis*, neomales (ZW females sex reversed into males) with Z chromosomes inherited from high-temperature-exposed sires (Shao et al., 2014). In the European sea bass, *Dicentrarchus labrax*, we found genes with methylation levels that resembled those of oocytes, while other genes had methylation levels resembling those of the sperm, suggesting female- and male-specific inheritance, respectively (Anastasiadi et al., 2018b).

**Figure 1:**
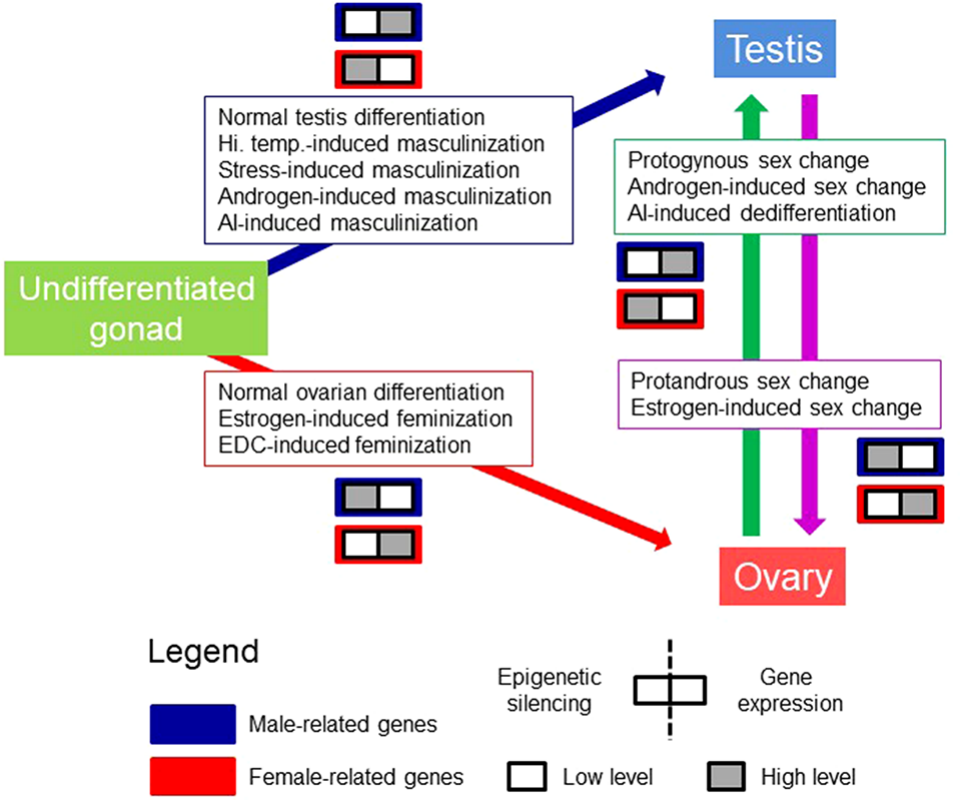
The model of Conserved Epigenetic Regulation of Sex (CERS). This model deals with the relationship between gene silencing features, e.g., DNA methylation, H3K9me enrichment, etc., and gene expression levels from an undifferentiated gonad to sex differentiation in the male (testis) and female (ovary) direction in gonochoristic species. It also contemplates the sex change in sequential hermaphrodite fishes. Pro-male (boxes with blue frame) and pro-female (boxes with red frame) genes refer to genes that are exclusively or preferentially expressed in one sex with respect to the other. In each box, the left half refers to epigenetic silencing, and the right half to gene expression levels. White and gray squares indicate lower and higher levels, respectively, of epigenetic silencing and gene expression. Boxed text indicates possible different means to arrive to a given phenotype. There might be other means. AI, aromatase inhibitor; EDC, endocrine disrupting chemical; Hi. Temp., high temperature. Figure modified from Piferrer (2019), with permission.

Testis development, at least in fish, where sex can be labile, can be achieved as a consequence of normal male sex differentiation, protogynous sex change, or as masculinization induced by high temperature, stress, aromatase inhibitors, or androgens (Blazquez et al., 2001; Navarro-Martin et al., 2009; Piferrer, 2019). Conversely, ovarian development can be achieved as a consequence of normal female sex differentiation, protandrous sex change, or feminization induced by estrogens or endocrine disrupting chemicals. The model is called conserved because the underlying mechanisms are thought to be shared across species even if they have different reproductive strategies (**Figure 1**). It should be noted that DNA methylation patterns may differ depending on the cell type within the same gonad. Thus, DNA methylation values reported until now in the gonads represent the combined values of the different cell types.

In the first inception of this model, the following aspects were discussed (Piferrer, 2019): 1) What species are more fruitful to study and why; 2) Which are the best developmental stages to target; 3) Whether there are other organs than the gonads worth targeting; 4) The links with ecotoxicology; and 5) The added comparative value of these studies. In this review, the concept CERS will be further developed. Thus, here we will: 1) Discuss some general considerations about epigenetic marks to put CERS and the concept of EEM in a broader perspective; 2) Since, in the last 2–3 years, several studies have provided information on DNA methylation levels and given the extraordinary diversity of fishes, we will attempt to summarize the available data on the epigenetic regulation of sex and hence test CERS. This will allow drawing conclusions that can be used not only to establish an appropriate framework but also to help to focus future studies; and 3) Make the suggestion that the CERS can be also applied to other vertebrates regardless of the sex-determining system, whether is genetic or environmental. In fact, even in plants, there is evidence of the involvement of epigenetic regulatory mechanisms in sex determination. This is the case of the *Populus balsamifera* tree, where the *pbrr9* gene showed sex-specific patterns of DNA methylation (mostly male-biased) in the putative promoter and in the first intron (Bräutigam et al., 2017).

## Epigenetic Biomarkers

### General Concepts

Biomarkers have been developed mostly in the context of human health (e.g., Liu et al., 2019). According to the Biomarkers Definitions Working Group, a biomarker is defined as “a characteristic that is objectively measured and evaluated as indicator of normal biological processes, pathogenic processes or pharmacologic responses to a therapeutic intervention” (Atkinson et al., 2001). Biomarkers can be proteins, levels of mRNA transcripts, or epigenetic modifications and can mainly be used for diagnosis and prognosis, e.g., to predict responses to therapy in cancer (Costa-Pinheiro et al., 2015; Prensner et al., 2012). Proper biomarkers have to be harmless and characterized by high sensitivity, specificity, and reproducibility (Atkinson et al., 2001; Costa-Pinheiro et al., 2015; García-Giménez et al., 2017).

Also in the context of human health, epigenetic alterations including DNA methylation, histone modifications, and noncoding RNAs have been suggested as good candidates for becoming cancer biomarkers because they can be stable, frequent, abundant, and accessible (Costa-Pinheiro et al., 2015). Nevertheless, the most frequently studied epigenetic modification as potential biomarker is DNA methylation, mainly because of its stability and relative ease of measurement by the available technologies (Bock, 2009; Van Neste et al., 2012). Thus, DNA methylation biomarkers are thought to be extremely promising in the context of human health (Van Neste et al., 2012; Costa-Pinheiro et al., 2015). However, other biomarkers such as microRNAs have been identified also as good candidates for human diseases (Navickas et al., 2016) and, to a lesser extent, as an aid in animal breeding programs (Ibeagha-Awemu and Zhao, 2015).

### Biomarker Development

**Figure 2:**
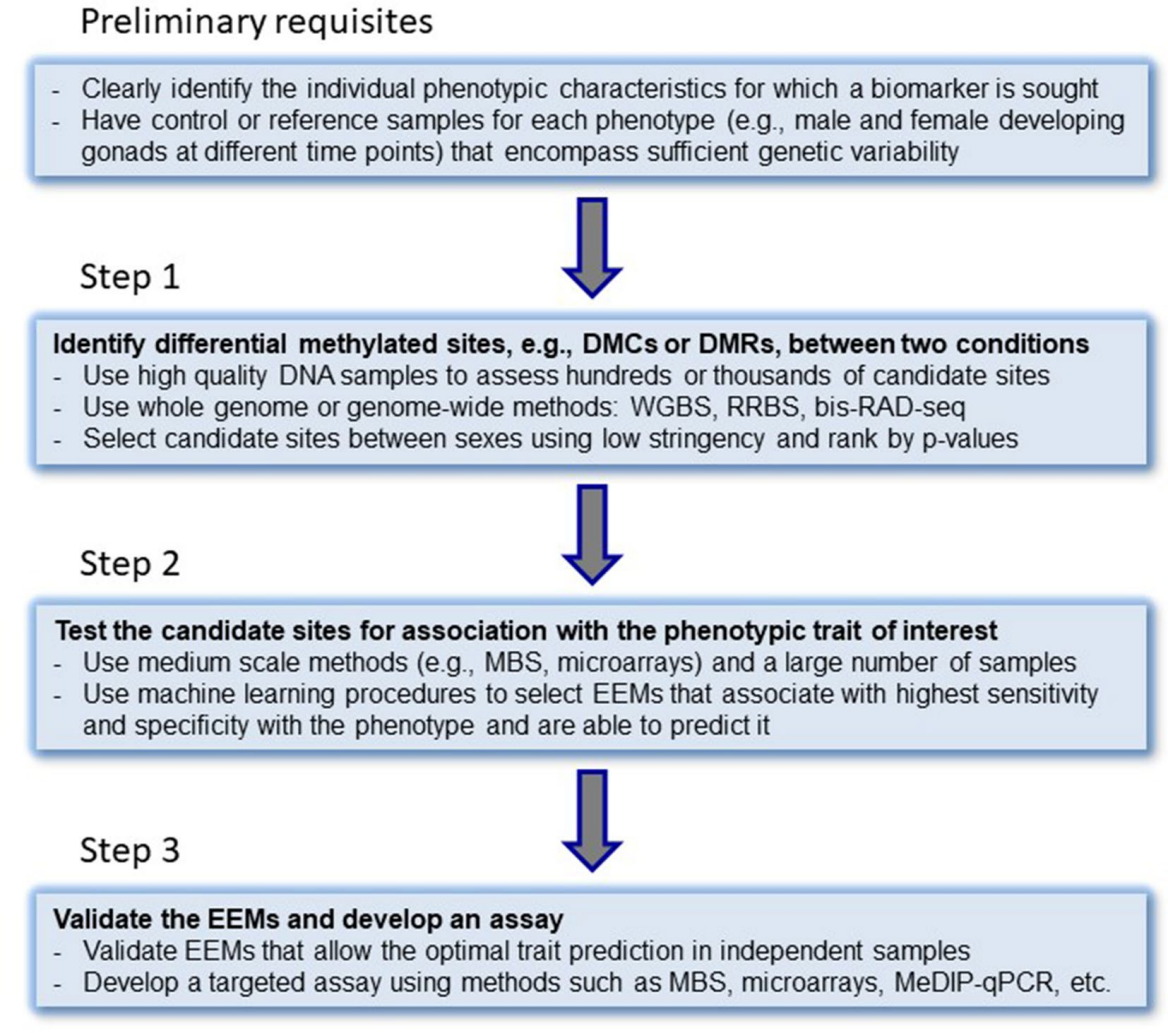
Step-by-step approach for the development of epigenetic biomarkers. DMCs, differentially methylated cytosines; DMRs, differentially methylated regions; WGBS, whole-genome bisulfite sequencing; RRBS, reduced representation bisulfite sequencing; bis-RAD-seq, bisulfite randomly amplified DNA sequencing; MBS, multiplex bisulfite sequencing; EEMs, essential epigenetic marks. MeDIP-seq, methylated DNA immunoprecipitation sequencing. The original idea of steps to epigenetic biomarker development was laid down by Bock (2009).

A systematic approach to develop epigenetic biomarkers based on DNA methylation has been suggested by Bock (2009) in the context of clinical applications, where different steps have to be completed. Here, we modify this approach for the development of epigenetic biomarkers to test the CERS (**Figure 2**). In the first step, a whole-genome or genome-wide method should be used in order to simultaneously assess hundreds or thousands of candidate sites. For DNA methylation biomarkers, whole-genome bisulfite sequencing (WGBS), reduced representation bisulfite sequencing (RRBS) (Gu et al., 2011), or bisulfite RAD-seq (Trucchi et al., 2016) could be employed. These techniques allow to measure the actual DNA methylation levels present in those cytosines located in a CpG context in vertebrate genomes and should lead to the identification of candidate EEMs. These can include differentially methylated cytosines (DMCs) or differentially methylated regions (DMRs) between sexes. In the second step, selected biomarkers are tested using targeted approaches in a large number of independent samples. Here, appropriate approaches include, but are not limited to, multiplex bisulfite sequencing (MBS) (Masser et al., 2013; Anastasiadi et al., 2018b), enrichment bisulfite sequencing (Diep et al., 2012; Paul et al., 2014), pyrosequencing, or mass spectrometric analysis of DNA methylation (Coolen et al., 2007; Bock et al., 2016). Computational and statistical, machine learning procedures involving regression (best subsets regression, penalized regression, principal components-based regression analysis) or classification analysis should be used. In the third step, from all those EEMs that are strongly correlated to the trait of interest, a handful of them that allow an optimal trait association and/or prediction are validated and a targeted assay is developed (array-type, MeDIP-qPCR or MBS) (Bøvelstad et al., 2007; James et al., 2013; Anastasiadi et al., 2018b).

Switching the perspective from clinical research to ecology and animal production, biomarkers in vertebrates have been used as indicators of environmental pollution (Monserrat et al., 2007) and animal health, including endocrine, immune, nutritional, and metabolic processes (Warne et al., 2015). Epigenetic biomarkers have already been used to predict age and sex in vertebrates. Thus, after the discovery of an epigenetic clock in humans, i.e., a panel of DNA methylation biomarkers as diagnostic of biological age (Horvath, 2013), epigenetic clocks have been constructed in other vertebrates, such as mice, *Mus muculus* (Han et al., 2018), chimpanzees, *Pan troglodytes* (Horvath, 2013), humpback whales, *Megaptera novaeangliae* (Polanowski et al., 2014), in a long-lived seabird, *Ardenna tenuirostris* (De Paoli‐Iseppi et al., 2019) and in the European sea bass (Anastasiadi et al., 2019).

### Development of Biomarkers of Sex

In livestock and animal production, epigenetic biomarkers have been suggested recently as candidates with extreme potential to predict the phenotypic outcome, as well as to improve production traits (Ibeagha-Awemu and Zhao, 2015; Moghadam et al., 2015). This need was first described in a report by the Food and Agriculture Organization of the United Nations in 2015 stating that the knowledge on epigenetics will offer new opportunities for animal breeding (Scherf and Pilling, 2015). In fish, using the European sea bass, as a model, a carefully selected panel of CpGs in three genes constitute an example of EEMs that were capable to predict the sex phenotype of the gonad with ∼90% accuracy (Anastasiadi et al., 2018b). To our knowledge, this is so far the first and only method to predict sex based on EEMs. Currently, sex prediction using EEMs is lethal and is not cost-effective. However, we are testing the possible existence of correlations between DNA methylation in predictor CpGs in the gonads with equally predictive CpGs in other tissues. On the other hand, the own development of biomarkers involves, in the last step, the use of CpG in an array-type approach or in multiplexing (MBS) that, along with the continued decrease of next-generation sequencing costs, should make the cost of screening per sample affordable.

## Testing the Model of the Conserved Epigenetic Regulation of Sex

Epigenetic regulation of gene expression is involved in the sexual development of gonochoristic fish with different types of sex-determining mechanisms, as well as in driving the process of sex change in different types of hermaphrodites.

**Figure 3:**
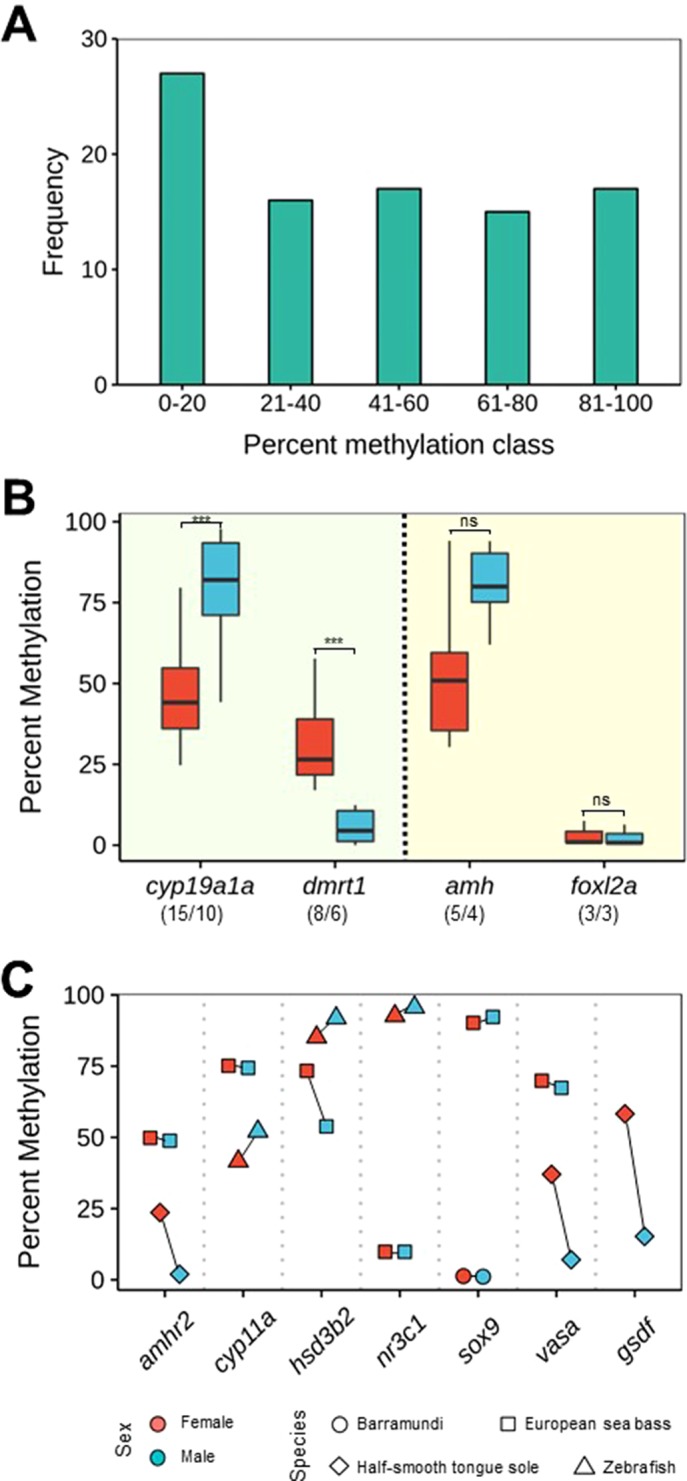
DNA methylation levels of some key genes (see **Table 1**) involved in sexual development. **(A)** Histogram of overall methylation levels for the genes discussed in this paper. Frequency refers to number of DNA methylation values obtained by combining published data and unpublished research performed in our lab. **(B)** Boxplot of DNA methylation levels of *cyp19a1a* and *dmrt1*, which conform to CERS postulates (left side), and *amh* and *foxl2*, which do not conform to CERS postulates (right side). The lower and upper hinges display the distribution of values between the first and third quartiles, the upper whisker extends to the maximum value up to 1.5 * interquartile range (IQR), the lower whisker extends to the minimum value up to 1.5 * IQR, while the black line indicates the median of the distribution. One outlier outside the end of the whiskers has been excluded. Numbers between parentheses indicate number of datapoints/species. If the first number is bigger than the second, it indicates that there are species for which there is more than one datapoint. Significant differences were assessed with the *t*-test. ***P < 0.001; ns, not significant. **(C)** DNA methylation levels of *amhr2, cyp11a, hsd3b2, nr3c1, sox9, vasa,* and *gsdf* in different species. For easier visualization, lines connect datapoints of the same species. In all genes except *gsdf*, there is data for at least two different species. In addition, in **(B** and **C)**, data are also color-coded according to sex.

Here, we searched the published literature in fish and collected information on the DNA methylation of genes related to sexual development. A WGBS was used in the half-smooth tongue sole, (Shao et al., 2014), while a MBS was used in the European sea bass (Anastasiadi et al., 2018b). These are the exceptions because in the rest of studies carried so far, which concern around 15 different species, just one or two genes have been analyzed in each case (**Table 1**). DNA methylation at a single CpG is of a binary nature, since a given CpG can be either methylated or unmethylated. However, mean percent DNA methylation can, theoretically, fall in any value between 0 and 100%. This applies regardless of whether one considers the promoter or the first intron (Anastasiadi et al., 2018a) or other genomic features in a predefined window of a given length. Information drawn from the primary literature shows that DNA methylation levels are more or less evenly distributed across five arbitrarily defined methylation classes (0–20%, 21–40%, 41–60%, 61–80%, and 81–100%), perhaps with a higher preponderance in the 0–20% class, regardless of other considerations such as method of analysis, targeted genomic feature, sex, species, etc. Thus, these preliminary data indicate that there are no preferred or typical DNA methylation values for the sex-related genes as a whole (**Figure 3A**). Again, it should be remembered that DNA methylation values represent the combined values resulting from the different cell types making up the gonads. Thus, the correlation with gene expression, if present, should take this into account.

**Table 1:** Studies involving fish where DNA methylation of genes associated with sexual development has been measured

Sex determination Species	Common name	Genes	References
***Gonochorism***			
*Dicentrarchus labrax* (Polygenic)	European sea bass	*cyp19a1a*	Navarro-Martín et al., 2011
		*amhr2, cyp19a1a, dmrt1, foxl2a, fshr, erβ2, nr3c1*	Anastasiadi et al., 2018b
		amh, cyp11a1, hsd3b2, sox9a, vasa (*)	Anastasiadi and Piferrer, 2019 (submitted)
*Danio rerio* (Polygenic)	Zebrafish	*amh*	Laing et al., 2018
		*amh, cyp11a1, cyp11c1, cyp19a1, dmrt1, foxl2a, hsd11β2, hsd17β1, hsd3b2, nr3c1*	Valdivieso et al., (unpublished)
		*cyp11c1, cyp19a1a, dmrt1, hsd11β2, hsd17β1*	Moraleda-Prados et al., (unpublished)
*Cynoglossus semilaevis* (ZW/ZZ)	Half-smooth tongue sole	amh, amhr2, arx, cxcr4a, cyp19a1a, daz1, dmrt1, emx2, figla, gsdf, lhx9, pdgfrb, sdf1a, vasa, wt1a, wt1b (**)	Shao et al., 2014
		*gata4*	Liu et al., 2016a
		*wnt4a*	Hu et al., 2014
		*rspon1*	Liu et al., 2018
*Scopthalmus maximus* (ZW/ZZ)	Turbot	*smpiwil2*	Wang et al., 2017a
*Gobiocypris rarus* (XX/XY)	Chinese rare minnow	*cyp19a1a*	Liu et al., 2014
*Paralichthys olivaceus* (XX/XY)	Olive flounder	*cyp19a1a, dmrt1*	Wen et al., 2014
*Oreochromis niloticus* (XX/XY)	Nile tilapia	*cyp19a1a*	Chen et al., 2017
		*cyp19a1a*	Wang et al., 2017b
		*cyp19a1a*	Chen et al., 2018b
*Lateolabrax maculatus* (XX/XY)	Chinese sea perch	*cyp19a1a*	Chen et al., 2018a
*Culter alburnus* (???)	Topmouth culter	*dmrt1*	Jia et al., 2019
***Hermaphroditism***			
*Achanthopagrus schlegelii* (Protandry)	Black porgy	*cyp19a1a*	Wu et al., 2016
*Lates calcarifer* (Protandry)	Barramundi	*amh, cyp19a1a, dmrt1, foxl2a, sox8, sox9a*	Domingos et al., 2018
*Monopterus albus* (Protogyny)	Ricefield eel	*cyp19a1a*	Zhang et al., 2013
*Kryptolebias marmoratus* (Simultaneous)	Mangrove killfish	*cyp19a1a*	Ellison et al., 2015

(*) This is from a multiplex bisulfite sequencing analysis with a larger panel of genes. Here, a subset of the most sex-related genes is shown.

(**) This subset of genes showed differential methylation level between ovaries and testes and are taken from Supplementary Table 8 in Shao et al. (2014), where whole-genome bisulfite sequencing was used.

Gonadal aromatase (*cyp19a1a*) was the first gene shown to be under epigenetic regulation during sexual development in a vertebrate, the European sea bass (Navarro-Martín et al., 2011). This is not surprising because it is the only steroidogenic enzyme responsible for the balance between androgens and estrogens and because estrogens are needed for ovarian differentiation in all nonmammalian vertebrates (Guiguen et al., 2010). Since then, the DNA methylation of only few genes has been studied in more than two species: *cyp19a1a* just cited earlier (studied in 10 species), doublesex- and mab-3-related transcription factor 1 (*dmrt1*) (6 species), anti-Müllerian hormone or Müllerian-inhibiting hormone (*amh*) (4 species), and the member of the winged helix/forkhead group (*foxl2a*) (3 species) (**Figure 3B**). From the analysis of the published data and our own unpublished data, we found that mean DNA methylation levels of *cyp19a1a* were typically <50% in ovaries (mean: 46.2%, sd: 15.98) and >75% in testes (mean: 77.0%, sd: 24.89) (*t*-test: -4.0439; df = 28, *p* = 0.00037) in a fairly consistent manner across species (see list of species in **Table 1**). This finding was in accordance with the constitutive higher expression of *cyp19a1a* in ovaries when compared with testes (Piferrer and Blázquez, 2005; Guiguen et al., 2010). Likewise, mean DNA methylation levels of *dmrt1* were ∼30% in ovaries (mean: 32.54%, sd: 15.98) and <10% in testes (mean: 5.54%, sd: 5.21) (*t*-test: 4.54; df = 14, *p* = 0.00046), also in accordance with the higher constitutive expression of *dmrt1* in testes when compared with ovaries (Herpin and Schartl, 2011) (**Figure 3B**). Therefore, these two important genes for sex differentiation, which have been used as sex markers in some fish species, e.g., turbot, *Scophthalmus maximus* (Ribas et al., 2016), and medaka, *Oryzias latipes* (Herpin and Schartl, 2011), do indeed conform to the CERS predictions, since there is an inverse relationship between DNA methylation and gene expression with clear sex-specific differences.

This inverse relationship does not seem apparent when two other well-known genes with sex-biased expression in fish are considered: *amh* and *foxl2a* (**Figure 3B**). *Amh* is a member of the TGF-β superfamily of growth and differentiation factors involved in sex differentiation from mammals to fish (Piferrer and Guiguen, 2008). Relatively low and equal levels of *amh* expression are detected in gonads prior to the appearance of sex-specific differences. However, once sex differentiation is underway, higher *amh* levels are typically associated with testis differentiation in several species analyzed (reviewed in Pfennig et al., 2015). Here, we found that mean DNA methylation levels of *amh* were 54.05% (sd: 25.26) in ovaries and 80.24% (sd: 12.74) in testes, a difference that did not reach statistical significance with the data available so far (*t*-test: -2.07; df = 8, *p* = 0.07211). In the same way, *foxl2a* is expressed at higher levels in the ovary when compared with the testis (reviewed in Bertho et al., 2016), like *cyp19a1a*. On the other hand, *foxl2a* is actually one of the earliest transcriptional activators of *cyp19a1a* that co-localizes in the granulosa cells (Wang et al., 2004). However, DNA methylation levels were clearly not different (mean = 3.08% and sd = 3.88 in ovaries and mean = 2.59% and sd = 3.3 in testes) (*t*-test: 0.1656; df = 4, *p* = 0.8765). Therefore, unlike *cyp19a1a* and *dmrt1*, and with the information available so far, data suggest that *amh* and *foxl2a* do not seem to conform to CERS predictions or that, in these genes, the relationship between DNA methylation and gene expression is positive (**Figure 3B**), although, clearly, further research is needed.

There are other genes related to sex differentiation at different degrees for which it may be premature to attempt any sort of generalizations. These genes include *amhr2*, *cyp11a*, *hsd3b2*, *nr3c1*, *sox9*, *vasa*, and *gsdf* (**Figure 3C**). Here, it is worth noting that allelic diversification of *amhr2* in *Takigugu rubripes* results in a dominant master sex-determining gene, while allelic diversification of *gsdf* has given rise to the sex-determining gene in some fish species, including *Oryzias luzonensis* and *Anoplopoma fimbria* (reviewed in Piferrer, 2018; Guiguen et al., 2019). DNA methylation levels of *amhr2* in the European sea bass were ∼50% without sex-related differences (Anastasiadi et al., 2018b), while in the half-smooth tongue sole DNA methylation levels are higher in females (Shao et al., 2014). Similarly, in the latter species, the only species where *gsdf* DNA methylation values have been determined, these values are clearly lower in males, in accordance with the higher expression of *gsdf* in males (Shao et al., 2014) (**Figure 3C**).

**Figure 4:**
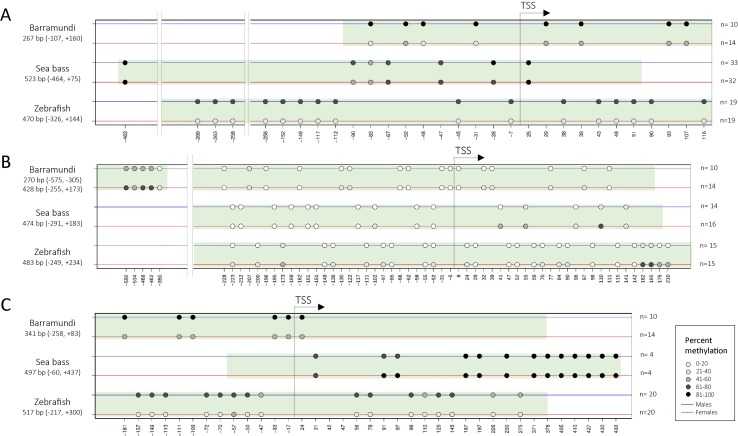
Representation of sex-specific differences in the DNA methylation of CpGs, indicated by circles, around the transcription start site of *cyp19a*1 **(A)**, *dmrt1* **(B)**, and *amh* **(C)** in three different species: Barramundi (Domingos et al., 2018), European sea bass (Anastasiadi et al., 2018b and our own unpublished data), and zebrafish (Valdivieso et al. unpubl. data). The shaded green area indicates the region targeted by the amplicon. Percent methylation is indicated by a gray scale. n = sample size. The graph was built with “Methylation plotter,” developed by Mallona et al. (2014) and available from http://maplab.imppc.org/methylation_plotter/

Except in the half-smooth tongue sole (Shao et al., 2014), where WGBS was used, in the rest of the studies reported in **Table 1** and used to draw **Figure 3**, targeted approaches were utilized to query the DNA methylation status of the target genes. For these studies, an average of ∼9 late juvenile or adult fish per sex was used. Typically, amplicons spawn ∼450 bp and usually include ∼15 CpG located around the transcription start site, although the latter figure may vary considerably among species. It is interesting to note that while sex-specific differences involve change in DNA methylation of several CpGs in some genes, in contrast, in other genes, sex-differences involve only a low number of CpGs (**Figure 4**). A more comprehensive picture will emerge when genome-wide DNA methylation techniques such as WGBS or RRBS will be employed in lieu of the targeted approaches used so far in most studies.

For the rest of the genes, *cyp11a*, *hsd3b2*, and *nr3c1*, there are only preliminary data gathered in our lab with the European sea bass (Anastasiadi et al., unpublished) and zebrafish (*Danio rerio*) (Valdivieso et al., unpublished; Moraleda-Prados et al., unpublished). In the European sea bass, methylation values of *hsd3b2* are higher in females. This is in agreement with the expression of this gene that is male-skewed in the developing gonads of Nile tilapia, *Oreochromis niloticus* (Ijiri et al., 2008). On the other hand, DNA methylation values of *nr3c1* and *sox9* were quite different between the two species.

In many species, sex determination has an environmental component. Hence, it is worth mentioning that an environmental factor such as temperature or population density may be connected to sex through epigenetic mechanisms. DNA methylation changes in sex-related genes is the type of epigenetic modification most commonly studied so far. Temperature can affect DNA methylation of many genes, as shown by MeDIP-seq in the Nile tilapia (Sun et al., 2016), although the exact mechanism is not known yet. In the European sea bass, elevated temperature induces hypermethylation in the promoter of *cyp19a1a*, and this prevents the binding of *cyp19a1a* transcriptional activators such as *sf1* and *foxl2a* (Navarro-Martín et al., 2011). Other epigenetic modifications can also be involved in the connection between environmental factors and sex. Thus, temperature increases the transcription of lysine-specific demethylase 6B (*kdm6b*), a chromatin modifier gene in the red-eared slider turtle, *Trachemys scripta*. *Kdm6b* eliminates the trimethylation of H3K27 in the promoter of *dmrt1*, leading to upregulation of its expression and male development (Ge et al., 2018; Georges and Holleley, 2018).

We would like to mention three considerations for further testing the CERS model. First, what species are worth testing? Obviously, fish, due to their great diversity of sexual systems and sex determining systems, which can vary even in closely related species. Reptiles can also provide very relevant information. Many reptiles possess temperature-dependent sex determination and thus offer the opportunity to test whether DNA methylation in key genes do correlate with gene expression and phenotypic sex under different incubation temperatures during the thermosensitive period. Thus, in the red-eared slider turtle *cyp19a1* DNA methylation levels conformed to CERS predictions (Matsumoto et al., 2013). The same is true in the alligator, *Alligator mississippiensis*, for *cyp19a1* and *sox9* (Parrott et al., 2014) and in the sea turtle, *Lepidochelys olivacea*, for *sox9* (Venegas et al., 2016). In birds and mammals, sexual development is strongly canalized (Capel, 2017), and therefore there is little or no room for sexual plasticity. Nevertheless, in such canalized systems, it would also be interesting to determine to what extent DNA methylation of key genes correlates with expression and whether this is established before the completion of gonadal differentiation. In any case, and regardless of the species of choice, testing the role of epigenetic regulation on the expression of key sex-related genes during the process of sex differentiation should involve, in our opinion, the analysis of at least three different time points. The first one, ideally, should be prior to any morphological sign of sex differentiation, the second around the middle of the process, and the third towards the end or after the completion of sex differentiation.

Second, what other genes can be targeted? In our view, the genes to be tested should include at least the ones that consistently follow or not the predictions of the CERS model, namely, *cyp19a1a*, *dmrt1*, *amh*, and *foxl2a*, as shown in this paper. However, other genes with known functions in sexual development in vertebrates, including mammals, birds, and reptiles, should also be studied. We propose here a list of some of the most relevant genes found in the literature (**Table 2**). Information on DNA methylation of additional genes during gonadal differentiation and any possible sex-related differences will help to better understand the epigenetic regulation of sexual development.

**Table 2:** Genes related to sexual development in mammals, birds, reptiles, and fish (Ge et al., 2018; Capel, 2017; Valenzuela et al., 2019; Todd et al., 2019) where its epigenetic regulation would be worth studying

Gene symbol	Gene description	Gene symbol	Gene description
amh*	Anti-Müllerian Hormone	foxl2*	Forkhead Box L2
amhr2*	Anti-Müllerian Hormone Receptor Type 2	*fst*	Follistatin
*crebp*	cAMP-Response Element-Binding Protein	gata4*	GATA Binding Protein 4
*ctnnb*	Beta-Catenin	gsdf*	Gonadal Soma Derived Factor
cyp11a*	Cytochrome P450 Family 11 Subfamily A Member 1	hsd11b2*	Hydroxysteroid 11-Beta Dehydrogenase 2
cyp11c1*	Cytochrome P450 Family 11 Subfamily B Member 1	*kdm6*	Lysine-specific Demethylase 6B
cyp19a1a*	Cytochrome P450 Family 19 Subfamily A Member 1	nr3c1*	Nuclear Receptor Subfamily 3 Group C Member 1
*dax1*	Nuclear Receptor Subfamily 0 Group B Member 1	rspo1*	R-Spondin 1
ddx4* (vasa)	DEAD-Box Helicase 4	*sf1*	Splicing Factor 1
dmrt1*	Doublesex And Mab-3 Related Transcription Factor 1	*sox17*	SRY-Box 17
erb2*	Estrogen Receptor Beta 2	sox9*	SRY-Box 9
*fgf9*	Fibroblast Growth Factor 9	*sry*	Sex Determining Region Y
figlα*	Folliculogenesis Specific BHLH Transcription Factor	wnt4*	Wnt Family Member 4
*fog2*	Zinc Finger Protein, FOG Family Member 2	wt1*	Wilms Tumor Protein 1

(*)Genes for which there is data on DNA methylation during sex differentiation, as detailed in **Table 1**.

Third, what other approaches can be used? Gene-editing techniques such as CRISPR/Cas9 or the more recently developed technique to edit the methylome in the mammalian genome by Liu et al. (2016b) can be very useful. To date, knockout mutants of sex-related genes in fish have been mostly developed for some model species, e.g., in zebrafish: *cyp19a1a* (Lau et al., 2016), *amh*, and *dmrt1* (Lin et al., 2017), and in medaka: estrogen receptor 1 (*esr1*) (Tohyama et al., 2017), *gnrh* family genes (Marvel et al., 2018), and *cyp19a1a* knockout (Nakamoto et al., 2018). Lau et al. (2016) found that all knockout mutants of *cyp19a1a* were males, supporting the view that aromatase plays an essential role in ovarian differentiation and development. Yet, Lin et al. (2017) found that *dmrt1* and *amh* knockout zebrafish mutants displayed female-biased sex ratios, but the development of abnormal testes was still possible. *Dmrt1* was suggested to be necessary for the maintenance, self-renewal, and differentiation of male germ cells, and *amh* was proposed to control the balance between proliferation and differentiation of these cells. Therefore, it would be interesting to analyze the DNA methylation of *dmrt1* and other male-biased genes in *amh* knockout mutants and vice versa, the DNA methylation of *amh* and other male-biased genes of the network in *dmrt1* knockout mutants.

## Gaps in Knowledge and Future Prospects

There are some aspects worth discussing regarding future studies of the involvement of DNA methylation on the regulation of sexual development. First, one aspect concerns the genomic feature on which one should focus when the goal is to associate DNA methylation with gene expression levels. Determination of the expression should be accurate and consistent for each gene assuming that the method of measurement, e.g., qPCR, is properly employed according to the appropriate standards (primers, reference genes, etc.). In contrast, DNA methylation levels can vary across different genomic features of the same gene. In most studies, the promoter region has been typically targeted. However, methylation of other genomic regions has been found to be equally or even better associated with gene silencing. Indeed, it was shown that the first exon is tightly linked to transcriptional silencing (Brenet et al., 2011). Furthermore, in a systematic study aimed at addressing this question, it was found that the first intron, more than the promoter and the first exon, is tightly related to gene silencing. This seems to be conserved across vertebrate species since it was observed in fish (*Japanese puffer*, *Takifugu rubipres*, and the European sea bass), frog (*Xenopus*), and humans (Anastasiadi et al., 2018a). Thus, for the epigenetic regulation of sex, as well as for sex-related development of biomarkers, it is better to focus around the transcription start site and to prioritize the CpGs localized in the first intron, first exon, and promoter regions, in the order mentioned. Furthermore, gene expression can also be positively correlated to tissue-specific DNA methylation, and this should be kept in mind (Lokk et al., 2014; Wan et al., 2015; Anastasiadi et al., 2018a).

Another aspect concerns the possible effect of genetic variation on DNA methylation levels and how to account for it in the data analysis (Lea et al., 2017; Anastasiadi et al., 2018b). This is related to the number of samples to be analyzed per treatment in studies of DNA methylation, which has been discussed elsewhere (Bock et al., 2016). Also, it would be desirable to overcome the noise induced by the cell heterogeneity of the gonadal tissue. In this regard, recent technological advances allow to determine the epigenome of single cells (Farlik et al., 2015). Efforts toward such type of measurements would definitively help in obtaining more robust measurements of DNA methylation.

Furthermore, DNA methylation and gene expression levels discussed throughout this paper refer to the gonads. DNA methylation is known to be tissue-specific. However, it cannot be ruled out that the methylation patterns of the gonads could be replicated in other tissues. This could be the case of tissues involved in the control of reproduction (e.g., the hypothalamus) or that present sex dimorphism (e.g., secondary sexual characters) because they are under the control of hormonal steroids. To the best of our knowledge, this information does not still exist despite increasing evidence of sex-related differences in DNA methylation for many genes in nonreproductive tissues, such as the muscle or the liver (Davegårdh et al., 2019; Grimm et al., 2019).

Another major challenge will be to determine the sexual phenotype just by the DNA methylation levels of selected EEMs before it can be determined by other means (e.g., by analyzing transcriptomic or histological changes). This would be achievable if demonstrated that the epigenetic modifications precede changes in gene expression. In this case, the EEMs and the CERS model can be foreseen as having potentially useful applications. For example, a defined set of EEMs could be used to predict the sexual phenotype in species with marked sexual growth dimorphism (Parker, 1992; Wang et al., 2019). EEMs could allow to predict the sex ratio in a subsample of a clutch before gonadal differentiation. This would aid in the stock management and in the selection of future broodstock. The same principle could be applied in ornamental fish culture, where the secondary sexual characteristics of males make them usually more desirable than females (Piferrer and Lim, 1997). Another case would be to aid in selection of broodstock fish with a certain epigenetic profile that is suitable to withstand, for example, a masculinization environment due to elevated density or temperature. In reptiles, the use of EEMs combined with temperature manipulations could aid in the research toward our understanding of the underlying molecular mechanism of temperature-dependent sex determination.

Finally, epigenetic modifications can recapitulate past environmental influences (Turner, 2009; Vogt, 2017). Taking advantage of this, EEMs could help to determine whether animals in the wild were exposed to altered environmental conditions such as, for example, exposure to pollutants or elevated temperatures. These EEMs could therefore be useful in conservation programs aimed at determining the environmental hazards to which natural populations may have been previously exposed. As an example along these lines, Guillette et al. (2016) identified epigenetic biomarkers to assess the environmental exposures and health impacts on populations of alligators from lakes contaminated with endocrine-disrupting compounds. The effects of endocrine-disrupting compounds on DNA methylation in the field of aquatic toxicology and biodiversity conservation have recently been reviewed by Tubbs and McDonough, (2018). This approach would allow determining whether a wild population was subjected to a sex-altering condition in the past. To our knowledge, this type of applications has not been fully developed to date, but several studies have started to identify biomarkers with this aim in mind.

## Conclusions

1. There are genes such as *cyp19a1a* and *dmrt1* where DNA methylation and gene expression in developing gonads are not only sex-specific but also inversely correlated. Thus, these genes conform to CERS predictions. Although certainly more research is needed, it is tempting to speculate that similar results will be found when new species are tested.
2. There are genes such as *amh* where DNA methylation and gene expression in the developing gonads could be positively correlated, and this relationship seems to be fairly conserved across species. Thus, in principle, these genes do not conform to CERS predictions. Undoubtedly, research in additional species is needed, but results probably will be similar to what has been found until now.
3. The clear sex-related differences in DNA methylation observed for *cyp19a1a* and *dmrt1* (and also for *amh* if more data confirm the trend observed here) suggest that their combined values could be used as EEMs in a test to predict gonadal sex in fish. Such a test is already available for the European sea bass and species with close sequence similarity for these genes (Anastasiadi et al., 2018b).
4. There are other genes for which there is less information available on DNA methylation, but the data collected so far suggest that perhaps *amhr2, hsd3b2, gsdf*, and *vasa* could conform to CERS predictions. These and other genes involved in sexual development should be examined in additional species.
5. The CERS model can become a useful tool to better focus research in other species and can contribute to our understanding of the role of epigenetic modifications in the regulation of gene expression during sexual development. However, a major question to be answered is whether sex-related differences in DNA methylation (either positively or negatively correlated) are a cause or a consequence of concomitant sex-related differences in gene expression. Based on the properties of epigenetic modifications, it is tempting to speculate that the role of epigenetic modifications is first to regulate and then to stabilize gene expression during sexual development. In fact, during this process, both somatic and germ cells differentiate and acquire identity in response to interactions with each other or with the environment, and epigenetic regulatory mechanisms are involved in differentiation and acquisition of cell identity.

## Data Availability

All datasets analyzed for this study are included in the manuscript and the supplementary files.

## Author Contributions

FP conceived the study, supervised the collection of data, coined the concepts of EEM and CERS, and wrote the paper. DA developed the technique of MBS and wrote the paper. AV, NS-B, JM-P, and LR collected data on different species and wrote the paper. All authors approved the final version of the manuscript.

## Funding

This study was supported by the Spanish Ministry of Science grants AGL2016–787107-R “Epimark” to FP and AGL2015-73864-JIN “Ambisex” to LR. DA was supported by an Epimark contract, AV and NS-B were supported by Spanish government scholarships (BES-2014-069051 and BES-2017-079744, respectively); LR and JM-P were supported by Ambisex contracts.

## Conflict of Interest Statement

The authors declare that the research was conducted in the absence of any commercial or financial relationships that could be construed as a potential conflict of interest.
